# Diversity and Occurrence of Tick-Borne Pathogens in Small Ruminants and Their Ticks in Sardinia: Implications for Animal Health and Zoonotic Risk

**DOI:** 10.3390/vetsci13080769

**Published:** 2026-07-31

**Authors:** Valentina Chisu, Rosanna Zobba, Laura Giua, Cipriano Foxi, Piera Bianco, Giovanna Chessa, Giovanna Masala, Alberto Alberti, Pierangela Cabras

**Affiliations:** 1 Istituto Zooprofilattico Sperimentale “G. Pegreffi” Della Sardegna, Via Duca Degli Abruzzi 8, 07100 Sassari, Italy; valentina.chisu@izs-sardegna.it (V.C.); lauragiua@live.it (L.G.); cipriano.foxi@izs-sardegna.it (C.F.); piera.bianco@izs-sardegna.it (P.B.); giovanna.chessa@izs-sardegna.it (G.C.); giovanna.masala@izs-sardegna.it (G.M.); pierangela.cabras@izs-sardegna.it (P.C.); 2 Dipartimento di Medicina Veterinaria, Università degli Studi di Sassari, 07100 Sassari, Italy; zobba@uniss.it

**Keywords:** molecular epidemiology, small ruminants, ticks, tick-borne diseases, Sardinia, Italy, One Health

## Abstract

Tick-borne diseases are an increasing concern in Mediterranean livestock systems, where environmental conditions and extensive grazing favour interactions among hosts, vectors, and pathogens. This study investigated the occurrence and diversity of tick-borne pathogens in small ruminants and their associated ticks in Sardinia (Italy), with the aim of characterizing the occurrence and diversity of tick-borne pathogens in small ruminants and their associated ticks in Sardinia. A cross-sectional survey was conducted on 17 farms in central-eastern Sardinia. Blood samples from 75 animals (57 goats and 18 sheep) and 364 ticks collected from infested hosts were analyzed. Ticks were morphologically identified, and both ticks and blood samples were screened for major bacterial and protozoan pathogens using rt-PCR and conventional PCR assays, followed by sequencing. Among examined hosts, *Anaplasma* spp. DNA was detected in 28% of goats and 39% of sheep, whereas *Theileria ovis* was identified in 4% of goats and 78% of sheep. No animals tested positive for *Rickettsia* spp. or *Coxiella* spp. Tick analysis revealed a predominance of *Rhipicephalus sanguineus* and *Rhipicephalus bursa*, with several tick-borne microorganisms detected, including *Rickettsia* spp. (26.6%), *Coxiella* spp. (22.5%), *Anaplasma* spp. (17.6%), and piroplasms (11.8%). Most *Coxiella*-positive ticks carried DNA of the *Coxiella*-like endosymbiont *Candidatus* Coxiella mudrowiae, while only one tick was positive for *Coxiella burnetii*. Sequencing confirmed the presence of zoonotic species such as *Rickettsia massiliae*, *Rickettsia sibirica/barbarica* group, and *Rickettsia hoogstraalii*. These findings reveal a complex epidemiological scenario characterized by frequent detection of tick-borne microorganisms in ticks but limited detection in hosts. The presence of zoonotic agents highlights potential public health risks and supports the need for integrated surveillance and control strategies in Mediterranean pastoral systems.

## Simple Summary

Tick-borne diseases are an important concern for livestock farming because ticks can transmit microorganisms that affect animal health and may also represent a risk for humans. In Mediterranean areas, where sheep and goats often graze outdoors, contact between animals, ticks, and pathogens can favour the spread of these infections. This study investigated the presence and diversity of microorganisms transmitted by ticks in small ruminants and their ticks in Sardinia, Italy, to better understand local transmission patterns. Blood samples from sheep and goats, and ticks collected from infested animals, were examined to identify the microorganisms present. DNA from several microorganisms associated with animal and human diseases was detected in ticks. Some of these microorganisms were also found in animals, particularly those associated with tick-borne diseases in sheep and goats. The results show that DNA from a variety of microorganisms of potential veterinary and public health relevance can be detected in ticks from Sardinian pastoral areas, even when corresponding infections are not always detected in animals. These findings highlight the importance of regular monitoring of tick populations and livestock health to reduce the impact of tick-borne diseases and protect both animal production and public health.

## 1. Introduction

Tick-borne diseases (TBDs) are among the most important emerging infectious diseases affecting both human and animal health worldwide [1]. Their increasing incidence and expanding geographic distribution are driven by complex interactions among environmental, ecological, and anthropogenic factors, including climate change, habitat modification, globalization, wildlife movements, and changes in livestock management systems [2]. These drivers have favoured the expansion of tick populations into previously unsuitable areas and have increased the opportunities for contact among vectors, domestic animals, wildlife, and humans, resulting in a growing burden of tick-borne pathogens on a global scale [3,4].

In livestock, tick-borne pathogens are responsible for substantial economic losses due to decreased productivity, reduced milk and meat yield, reproductive disorders, increased veterinary costs, and mortality. In addition to their direct impact on animal health and welfare, these infections may compromise farm profitability and represent an important constraint to sustainable livestock production, particularly in regions where extensive grazing systems predominate [5]. Many tick-borne microorganisms are also zoonotic, highlighting the importance of adopting a One Health approach that integrates veterinary, medical, and environmental surveillance.

Small ruminants, particularly sheep and goats, are highly exposed to tick infestations due to their widespread management under extensive and semi-extensive systems. Continuous grazing and interactions with wildlife favour contact with tick habitats and contribute to the maintenance of pathogen transmission cycles. In endemic areas, repeated exposure to infected ticks may result in persistent or subclinical infections, supporting long-term pathogen circulation within livestock ecosystems [5]. Globally, tick-borne diseases represent a major constraint for sheep and goat production, with *Anaplasma*, *Babesia*, *Theileria*, *Ehrlichia*, *Rickettsia*, and *Coxiella* among the most relevant pathogens, causing economic losses through reduced productivity, reproductive disorders, treatment costs, and mortality [6,7].

The Mediterranean basin represents one of the European regions with the highest diversity of tick species and tick-borne pathogens. Its climatic conditions—characterized by mild winters and warm, dry summers—favour the development and survival of several Ixodidae ticks throughout much of the year. Among these, species belonging to the genus Rhipicephalus are particularly widespread and are recognized vectors of numerous bacterial and protozoan pathogens of veterinary and medical importance. Recent studies conducted in several Mediterranean countries have reported the circulation of *Anaplasma*, *Babesia*, *Theileria*, *Rickettsia*, *Ehrlichia*, and *Coxiella* species both in domestic animals and ticks, emphasizing the epidemiological complexity of these ecosystems [8].

Within this context, Sardinia represents a particularly interesting epidemiological setting. As the second largest island in the Mediterranean Sea, it combines favourable climatic conditions, extensive pastoral farming systems, abundant wildlife populations, and a remarkable diversity of tick species. The Sardinian zootechnical sector is largely based on ovine and caprine production, which contributes substantially to regional income and supports traditional agro-pastoral systems [9]. Beyond their economic importance, these production systems provide essential ecosystem services by maintaining open landscapes, preserving biodiversity, reducing shrub encroachment, and contributing to wildfire prevention [10]. However, the same ecological characteristics that support these traditional farming systems also facilitate the maintenance and circulation of ticks and tick-borne pathogens through continuous interactions among livestock, wildlife, companion animals, and humans [4,11]. Tick infestations and the pathogens they transmit represent an increasing epidemiological and economic concern for both livestock production and public health [6].

Sardinia is considered endemic for several tick-borne pathogens affecting both animals and humans, with a high diversity of microorganisms circulating among ticks and vertebrate hosts. Molecular studies have shown that *Rhipicephalus sanguineus* and *Rhipicephalus bursa* are widely distributed in the region and are associated with several pathogens of veterinary and zoonotic relevance. These include bacteria of the genera *Rickettsia*, *Anaplasma*, *Ehrlichia*, and *Coxiella*, as well as protozoa such as *Babesia* and *Theileria* [12,13,14].

The detection of multiple microorganisms in the tick population indicates a complex epidemiological scenario characterized by the potential co-occurrence of microorganisms and dynamic host–vector–pathogen interactions. Human cases of rickettsioses sensu lato have also been documented on the island, highlighting the zoonotic risk associated with local tick fauna [15,16].

Despite the growing number of studies describing the occurrence of tick-borne microorganisms in Sardinian ticks collected from humans and domestic and wild mammals [12,13,14], comparatively little information is available regarding the simultaneous investigation of pathogens in both small ruminants and the ticks parasitizing them. Analyzing hosts and vectors concurrently provides a more comprehensive understanding of local transmission dynamics and helps clarify the epidemiological relationships between the occurrence of tick-borne microorganisms in ticks and their detection in vertebrate hosts. Furthermore, most available studies have focused on single pathogens or single host species, whereas relatively few have explored the diversity of bacterial and protozoan agents circulating simultaneously within the same farming systems.

Understanding these complex host–vector–pathogen interactions is essential for designing effective surveillance programmes, improving tick control strategies, and reducing the risk of pathogen transmission to both livestock and humans. Such information is particularly relevant under a One Health framework, in which veterinary surveillance contributes directly to protecting public health and environmental sustainability.

Therefore, the present study aimed to investigate the occurrence and genetic diversity of major tick-borne bacterial and protozoan pathogens in sheep, goats, and their associated ticks collected from extensive farming systems in central-eastern Sardinia. By combining molecular detection with sequence analysis of pathogens identified in both vertebrate hosts and their ectoparasites, this study provides new insights into the epidemiology of tick-borne infections circulating in one of the principal pastoral regions of the Mediterranean basin, providing updated epidemiological data and discussing their implications for animal health and zoonotic risk.

## 2. Materials and Methods

### 2.1. Study Area

A cross-sectional study was carried out in 2025 in central-eastern Sardinia, Italy, mainly within the Ogliastra region but included additional areas in the Sarrabus-Gerrei subregion (Castiadas and Villaputzu municipalities). The Ogliastra region covers approximately 1855 km^2^ and includes 23 municipalities, with a total population of about 53,000 inhabitants [17]. Castiadas and Villaputzu were included due to their ecological continuity with Ogliastra and the presence of similar environmental and farming conditions.

The region is characterized by a high environmental value and remarkable ecological diversity. The region includes the mountainous landscapes of the Gennargentu massif, extensive forests, valleys, Mediterranean scrub dominated by species such as *Cistus* spp., *Pistacia lentiscus*, *Myrtus communis*, and *Rosmarinus officinalis*, as well as agricultural coastal plains, river systems, and rugged rocky shorelines. These heterogeneous environments support a rich wildlife community, including Sardinian mouflon, Sardinian red deer, wild boar, foxes, and numerous bird species. Livestock farming is one of the main rural activities and is predominantly based on traditional extensive grazing systems involving sheep, goats, cattle, pigs, and horses. Under these management conditions, domestic animals frequently share natural pastures with wildlife. These characteristics make Ogliastra and Sarrabus-Gerrei representative Mediterranean agro-pastoral systems for investigating tick infestation and tick-borne pathogen occurrence in small ruminants. The persistence of low-intensity traditional pastoral systems and the high degree of landscape naturalness distinguish this area from more intensively managed livestock regions [18].

### 2.2. Ethical Statement

All methods were carried out in accordance with relevant guidelines and regulations. The study did not involve any animal experiments. Sample collection was carried out during the routine clinical visits of sheep and goats by the veterinary practitioners in farms.

### 2.3. Farm Selection and Animal Sampling

**Figure 1:**
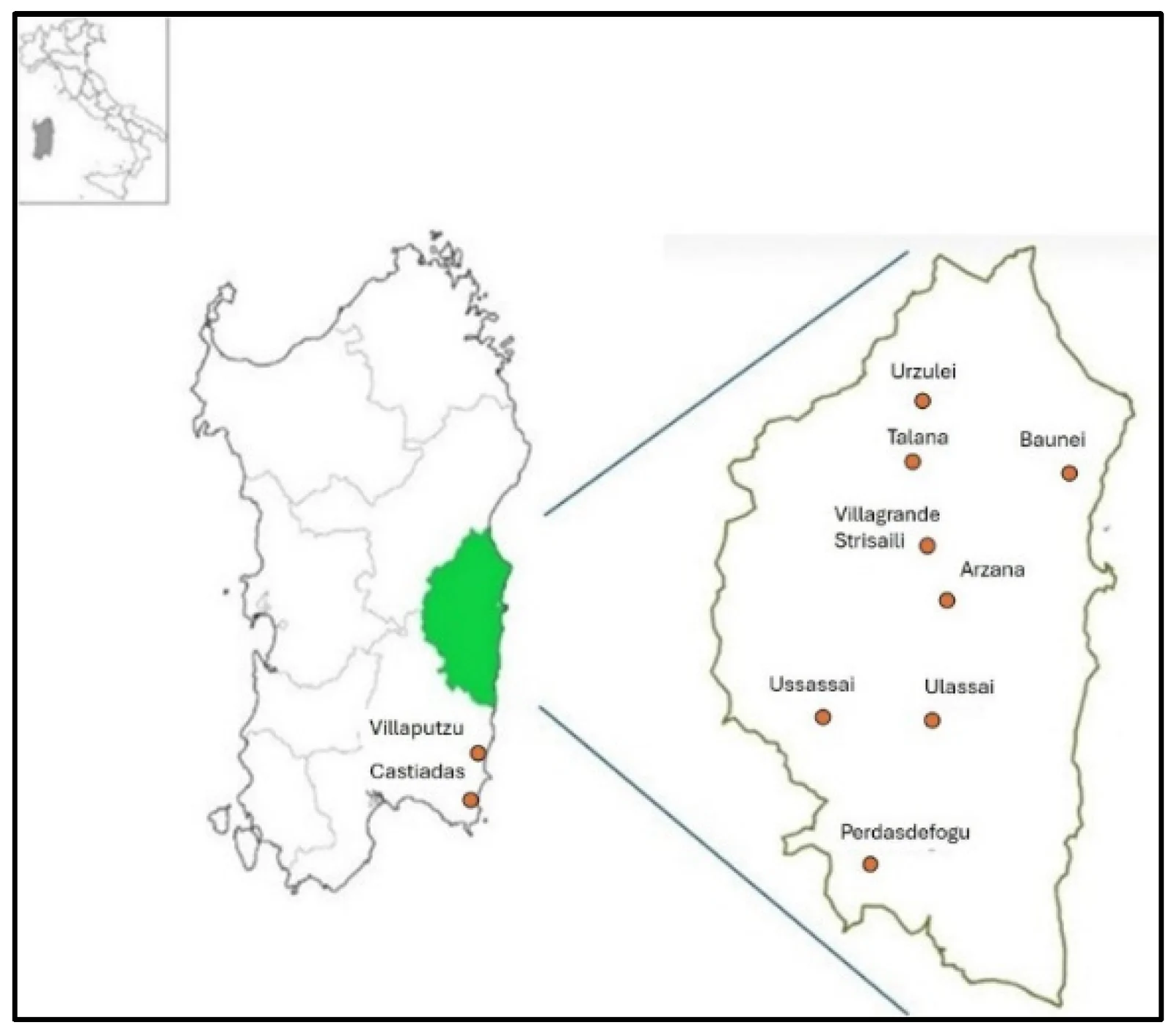
Location of the 17 livestock farms included in this study, distributed across 10 municipalities in central-eastern Sardinia, Italy.

Seventeen livestock farms distributed across 10 municipalities of the Ogliastra region (Baunei, Ussassai, Ulassai, Talana, Villagrande Strisaili, Urzulei, Perdasdefogu, Castiadas, and Villaputzu, Arzana) were included in the study (Figure 1). Sampling was conducted from April to October 2025, covering the period of tick activity in the study area.

Farms were selected based on accessibility, farmer consent, and geographic representativeness, covering various altitudes and ecological conditions. The selected farms raised goats and sheep, with herd sizes ranging from 26 to over 400 animals (Table 1). On each farm, animals were randomly selected among those available at the time of visit.

### 2.4. Clinical Examination and Data Collection

A total of 75 small ruminants (57 goats and 18 sheep) from 12 farms was sampled for blood collection. Additionally, 364 ticks were collected from a total of 86 animals (64 goats and 22 sheep), including the 74 out of the 75 blood-sampled animals and an additional 7 goats and 4 sheep examined exclusively for tick collection from five other farms. Ticks collected from the same animal and/or within the same farm were considered as potentially clustered observations, as multiple ticks may originate from the same host or farm environment. Therefore, tick data were interpreted descriptively to characterize tick species composition and the detection of tick-borne pathogens, without considering individual ticks as independent epidemiological units. All sampled animals underwent a clinical examination to assess their general health status, with particular attention paid to signs potentially associated with tick-borne infections, such as poor body condition, anemia, lymphadenopathy, and fever. Animals were systematically inspected for the presence of ticks, starting from the head and neck and proceeding caudally to the perineal and tail regions.

### 2.5. Sample Collection and Tick Identification

Whole blood samples were collected from the animals by jugular venipuncture into EDTA tubes for molecular analyses and stored at −20 °C until DNA extraction. Ticks were manually removed from animals using forceps, placed in sterile tubes, and transported to the laboratory of the Istituto Zooprofilattico Sperimentale of Sardinia for morphological identification and molecular analyses. Ticks were identified to species and developmental stage under a stereomicroscope using standard morphological keys [19].

### 2.6. DNA Extraction and Molecular Detection of Tick-Borne Pathogens

Genomic DNA was extracted from 100 µL of whole blood and from tick homogenates using a DNeasy Blood and Tissue Kit (QIAGEN, Hilden, Germany), following the manufacturer’s instructions. Each extraction batch included a negative control consisting of nuclease-free water to monitor potential contamination. Extracted DNA samples were screened for several vector-borne bacterial of veterinary and zoonotic relevance, including *Rickettsia* spp., *Anaplasma* spp., *Ehrlichia* spp., *Coxiella burnetii*, *Babesia* spp, and *Theileria* spp.

Real-time PCR assays were performed for tick DNA samples using bacterial-specific primers and probes, following previously published protocols (Table 2).

Each reaction was carried out in a final volume of 13 µL, containing 2.5 µL of 5× PCR master mix quantifast, 10 µM of each primer, 10 µM probe, and 3 µL of template DNA. Amplifications were performed in a real-time PCR system (7500 Real-Time PCR System, Applied Biosystems, Foster City, CA, USA) under the following conditions: an initial denaturation step at 95 °C for 5 min, followed by 45 cycles of denaturation at 95 °C for 15 s and annealing/extension at 60 °C for 30 s. Thermal cycling conditions were adjusted according to the specific target. Samples showing amplification signals with cycle threshold (Ct) values ≤45 were considered positive.

All samples testing positive by rt-PCR were further analyzed by conventional PCR assays to confirm bacterial identity. Conventional PCR reactions were performed in a final volume of 25 µL containing 12.5 µL of 2× PCR master mix, 25 µM of each primer, and 1 µL of template DNA (QuantiFast Pathogen RT-PCR +IC Kit, Qiagen, Hilden, Germany). Amplifications were carried out under the following cycling conditions: initial denaturation at 95 °C for 5 min, followed by 40 cycles of denaturation at 95 °C for 30 s, annealing at target-specific temperatures for 30 s, and extension at 72 °C for 60 s, with a final extension step at 72 °C for 5 min. PCR products were visualized by electrophoresis on a 1.5% agarose gel stained with SYBR Safe DNA Gel Stain (Invitrogen, Carlsbad, CA, USA, USA) and examined under UV illumination. All PCR assays included appropriate positive and negative controls. Samples were considered positive according to the amplification criteria of the respective assay.

### 2.7. Purification and Sequencing

All PCR amplicons were subjected to Sanger sequencing; however, only those yielding readable electropherograms were retained for subsequent analyses. When sequencing failed to generate a readable electropherogram, the sample was recorded as PCR-positive but remained unconfirmed by sequencing and was excluded from species-level identification.

Amplicons were purified using the QIAquick PCR Purification Kit (Qiagen, Hilden, Germany) according to the manufacturer’s protocol, and bidirectionally sequenced by DNA sequencing kit (dRhodamine Terminator cycle sequencing ready reaction; Applied Biosystems), according to the manufacturer’s instructions. Chromatograms were edited with Chromas 2.2 (Technelysium, Helensvale, Australia) and pairwise/multiple sequence alignments and sequence similarities were calculated using the ClustalW and the identity matrix options of Bioedit [27], respectively. Sequences obtained were then compared with those present in the GenBank database using the BLASTN search tool (v2.16.0; https://blast.ncbi.nlm.nih.gov; accessed on 1 December 2025).

All sequences generated in this study have been deposited in the GenBank database.

## 3. Results

### 3.1. Animals

DNA of *Rickettsia* spp. and *Coxiella* spp. was not detected in any of the 75 animals tested by PCR (57 goats and 18 sheep). In contrast, 16 of 57 (28%; 95% CI: 18.1–40.8%) goats and 7 of 18 (39%; 95% CI: 20.3–61.4%) sheep were PCR-positive for *Anaplasma* spp. Sequencing was attempted on 10 PCR-positive goat samples and one PCR-positive sheep sample. The obtained sequences were consistent with *Anaplasma* spp.; however, identical sequence matches were obtained for multiple species, preventing reliable species-level identification.

Sixteen animals (2/57 goats (4%; 95% CI: 1.0–11.9%) and 14/18 sheep (78%; 95% CI: 54.8–91.1%)) were PCR-positive for piroplasms. Sequencing was attempted for ten PCR-positive samples. All ten yielded sequences consistent with piroplasms, whereas only four generated chromatograms of sufficient quality to allow reliable species-level identification as *Theileria ovis* and deposition in GenBank (Table 3).

Sequencing of the two amplicons from goats and eight amplicons from sheep confirmed their identity as piroplasms. Among all the electropherograms obtained, only four—derived from sheep samples—were of sufficient quality to be deposited in GenBank and identified as *T. ovis* (Accession number: PZ256981–PZ256984).

### 3.2. Ticks

**Figure 2:**
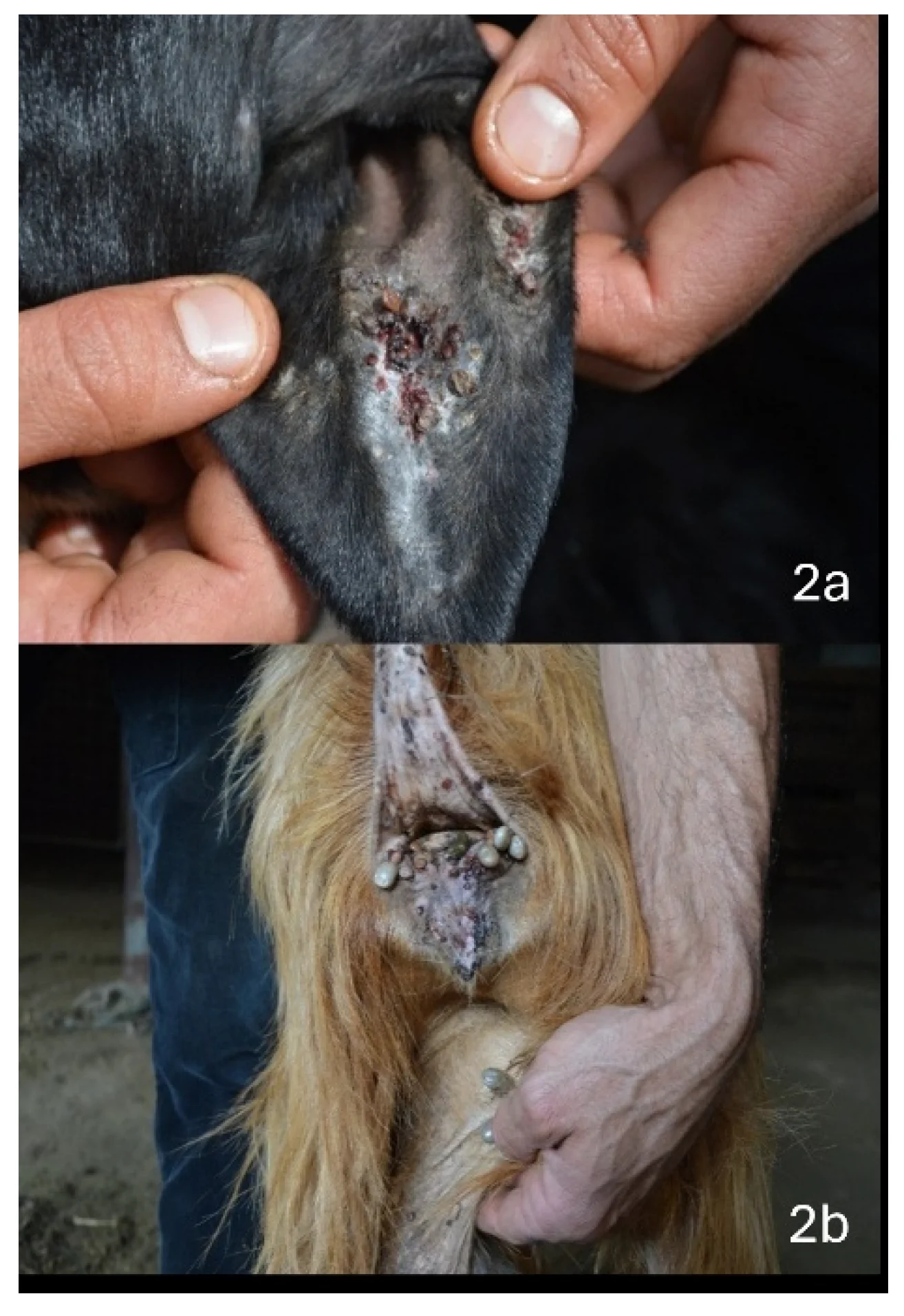
Representative tick infestations observed during clinical examination. (**a**) Tick infestation and associated lesions on the ear of a sheep. (**b**) Tick infestation in the perineal and udder regions of a goat.

The morphological characterization of the 364 ticks analyzed in this study revealed the presence of 251 *R. sanguineus*, 110 *R. bursa*, one *R. turanicus*, one *R. pusillus*, and one *Haemaphysalis sulcata*. Ticks were most frequently found on the head, ears, perineal and udder regions (Figure 2).

Among the 364 ticks tested, piroplasm DNA was detected in 43 ticks (11.8%; 95% CI: 8.9–15.5%), *Coxiella* spp. in 82 (22.5%; 95% CI: 18.5–27.1%), *Rickettsia* spp. in 94 (25.8%; 95% CI: 21.6–30.5%), and *Anaplasma* spp. in 67 (18.4%; 95% CI: 14.8–22.7%). Positive ticks were mainly identified as *R. sanguineus* and *R. bursa*, with sporadic detection in *H. sulcata*, *R. turanicus*, and *R. pusillus* (Table 4).

Among the 94 *Rickettsia* RT-PCR-positive samples, 42 were further analyzed by conventional PCR, of which 28 yielded positive results. Sequencing was successfully performed on 27 amplicons. Among these sequences, 19 (13 from Baunei and 6 from Ussassai) showed 100% identity with *Rickettsia sibirica/barbarica*, whereas 7 (5 from Baunei and 2 from Ussassai) showed 100% identity with *Rickettsia massiliae* (Accession numbers: PZ269681–PZ269706). All sequenced amplicons from these samples were obtained from ticks identified as *Rhipicephalus sanguineus*. Additionally, one sequence obtained from a *Hyalomma sulcata* tick collected from sheep in the Baunei farm showed 100% identity with *R. hoogstraalii* (Accession number: PZ269707).

Among the 82 *Coxiella* RT-PCR-positive samples, 79 were further analyzed by conventional PCR, and 55 yielded positive results. All 55 amplicons, obtained from *R. sanguineus* ticks collected from goats on the Baunei farm, were sequenced. Of these, 54 showed 100% identity with *Candidatus* Coxiella mudrowiae (Accession numbers: PZ256719–PZ256756), whereas one showed 100% identity with *Coxiella burnetii* (Accession number: PZ256718).

A detailed distribution of RT-PCR results according to farm and tick species is presented in Table 4.

Regarding the host–vector relationship, the co-detection of the same pathogen in animals and their associated ticks was observed only in a subset of cases. As shown in Table 5, among the 35 animals infested with *Anaplasma*-positive ticks, only 11 animals (8 goats and 3 sheep) were also positive for the same pathogen.

Similarly, Table 6 shows that among the 22 animals infested with piroplasm-positive ticks, only 7 animals (6 sheep and 1 goat) were also positive for piroplasms.

## 4. Discussion

This study provides a comprehensive overview of tick infestation and tick-borne microorganisms detected in small-ruminant farms in Sardinia, highlighting a multifaceted epidemiological context in which several tick species and multiple pathogens coexist within Mediterranean agro-pastoral ecosystems. The results confirm that small-ruminant production systems represent important ecological interfaces where domestic animals, arthropod vectors, environmental factors, and potentially wildlife reservoirs interact continuously [8,28]. In this context, surveillance of tick populations and associated microorganisms is essential not only for evaluating animal health risks but also for understanding potential implications for public health according to a One Health perspective [28].

The tick community identified in this study was dominated by species belonging to the genus *Rhipicephalus*, particularly *R. sanguineus* sensu lato and *R. bursa*, which accounted for the majority of the 364 collected specimens. The predominance of these tick species is consistent with previous investigations performed in Sardinia and other Mediterranean regions, where *Rhipicephalus* spp. is among the most abundant ticks infesting domestic ruminants [8,12,29,30]. Their ecological success is largely associated with their adaptation to warm climates, their ability to exploit domestic hosts, and their capacity to complete their life cycle in environments characterized by extensive livestock management systems [6,19].

The detection of occasional specimens belonging to *R. turanicus*, *R. pusillus*, and *H. sulcata* indicates that the tick fauna present in the investigated farms is more diverse than suggested by the dominance of *Rhipicephalus* species. Although these taxa were identified only sporadically, their presence reflects the ecological complexity of Sardinian rural landscapes, where livestock often share habitats with wild mammals that can act as alternative hosts for ticks [18]. Interactions between domestic animals and wildlife are increasingly recognized as important drivers of tick population dynamics and pathogen circulation, particularly in Mediterranean environments characterized by fragmented landscapes and extensive grazing systems [31].

The anatomical distribution of ticks observed in the present study was consistent with previous observations in small ruminants, where ixodid species show marked preferences for specific attachment sites according to their ecological adaptations and host–parasite interactions [32,33]. Ticks were mainly collected from the head, ears, udder, and perineal regions, which represent preferred attachment sites due to favourable humidity, reduced grooming efficiency, and thinner skin areas that facilitate blood feeding [34,35]. The localization of ticks on specific body regions may also influence pathogen transmission dynamics, as prolonged attachment and feeding duration increase the probability of successful pathogen exchange between vector and host [36].

The detection of multiple microorganisms, including *Anaplasma* spp., *T. ovis*, *Rickettsia* spp., and *Coxiella*-related bacteria, confirms that Sardinian tick populations harbour complex microbial communities rather than acting as simple carriers of individual pathogens [8,37]. This microbial diversity is particularly relevant in Mediterranean agro-pastoral ecosystems, where climatic conditions and extensive livestock farming promote interactions among ticks, domestic animals, wildlife, and humans, favouring the maintenance of diverse vector-associated microorganisms [8,38].

Sequencing identified *R. massiliae* and *R. sibirica/barbarica* in *R. sanguineus* ticks. Both species belong to the spotted fever group (SFG) rickettsiae and have been associated with human cases of spotted fever [39,40]. *R. sanguineus* sensu lato has been implicated in the epidemiology of *R. massiliae* [41]; however, the detection of *Rickettsia* DNA in field-collected ticks does not necessarily demonstrate vector competence or active pathogen transmission [42].

Their detection confirms the circulation of medically relevant *Rickettsia* species in pastoral environments and highlights the potential zoonotic implications of tick exposure in livestock settings [43,44].

Another finding was the frequent detection of *Ca.* Coxiella mudrowiae, a *Coxiella*-like endosymbiont commonly found in ticks, in specimens collected from farms in Baunei. Unlike *Coxiella burnetii*, the causative agent of Q fever, these endosymbionts are not considered pathogenic to vertebrates and are believed to contribute to tick physiology and reproduction [45,46]. The widespread presence of *Coxiella*-like endosymbionts has been reported in various tick species worldwide and may affect ticks’ vector competence for other pathogens [13,47] (Chisu et al., 2021; Rahal et al., 2020).

DNA of *Anaplasma* spp. (67/364; 18.4%) and *Theileria ovis* (43/364; 11.8%) was also detected in *Rhipicephalus* ticks. This finding supports the epidemiological association between the detection of these microorganisms in ticks and their detection in livestock from the same farms. However, the presence of pathogen DNA in ticks does not necessarily demonstrate their vector competence, as it may reflect residual blood from infected hosts.

While the analysis of ticks demonstrated the circulation of a diverse range of tick-borne microorganisms, molecular findings obtained from sampled small ruminants revealed a partially different epidemiological scenario. Specifically, blood sample analysis confirmed the circulation of *Anaplasma* spp. and *Theileria ovis* among the sampled animals, whereas a discrepancy was observed for the detection of *Rickettsia* spp. and *Coxiella* spp. Furthermore, the descriptive comparison between *Anaplasma*- and piroplasm-positive animals and their associated ticks showed that the detection of pathogen DNA in hosts did not always coincide with the presence of positive ticks collected from the same animals. However, these observations should be interpreted cautiously due to several limitations.

First, the detection of microbial DNA in ticks does not necessarily indicate the presence of viable pathogens or active transmission, as it may represent residual DNA acquired during previous blood meals [46]. Second, the presence of a pathogen in a tick does not imply vector competence, since successful pathogen transmission depends on complex interactions [48]. Finally, infections in vertebrate hosts are often transient, with periods of bacteraemia or parasitaemia that may be too short or occur at levels below the detection limit of PCR assays performed on peripheral blood. Consequently, infected animals may test negative despite previous or ongoing exposure [6]. Similar discrepancies between pathogen detection rates in ticks and in vertebrate hosts have been reported for several tick-borne pathogens, highlighting that pathogen circulation within vector populations does not necessarily translate into detectable infections in host populations [49,50].

The presence of *Anaplasma* spp. was observed in both goats and sheep, with positivity rates of 28% (16/57) and 39% (7/18), respectively. Although sequencing analysis confirmed the presence of *Anaplasma* DNA, the limited length of the obtained sequences prevented accurate species-level identification. Nevertheless, the results are consistent with previous studies reporting the circulation of *Anaplasma* species among Mediterranean livestock populations [14,51]. The epidemiological relevance of these findings is supported by the simultaneous detection of *Anaplasma* DNA in ticks collected from the same farms, indicating the presence of this bacterial genus within the local tick–host system.

In Mediterranean regions, *Anaplasma* infections in small ruminants are often characterized by subclinical or chronic forms, particularly in areas where animals are continuously exposed to ticks harbouring tick-borne microorganisms [52]. Under endemic conditions, repeated exposure may contribute to the development of acquired immunity, reducing the severity of clinical manifestations while allowing persistent circulation of the microorganism within animal populations [53]. The absence of reported clinical signs among positive animals in the present study may therefore reflect an endemic stability scenario, where infection occurs frequently but disease expression remains limited [54]. However, alternative explanations should also be considered. The detected *Anaplasma* strains may include species or variants with limited pathogenic potential in small ruminants. Indeed, the genus *Anaplasma* includes multiple species with different levels of host adaptation and pathogenicity, ranging from clinically relevant agents such as *A. phagocytophilum* to less pathogenic organisms frequently detected in livestock and ticks [55]. Additional sequencing approaches, including longer genomic targets or whole-genome analyses, would be useful to better characterize the Anaplasma strains circulating in Sardinian farms and evaluate their veterinary significance.

The detection of *Theileria ovis* in both goats and sheep confirms the circulation of this protozoan parasite in Sardinia [56]. Notably, prevalence varied substantially between host species, with only 4% (2/57) of goats testing positive compared to 78% (14/18) of sheep. This marked disparity is consistent with previous studies indicating that sheep are the primary domestic host for this parasite [57,58]. Unlike highly pathogenic *Theileria* species responsible for severe clinical disease in susceptible hosts, *T. ovis* is generally considered a low-pathogenicity parasite, with infections often remaining asymptomatic or causing mild hematological alterations [59,60]. Therefore, the identification of infected animals without apparent clinical abnormalities is not unexpected and suggests that this parasite may be widely established in Sardinian flocks without causing significant overt disease. Nevertheless, chronic infections may still have indirect consequences, particularly through potential effects on productivity, immune status, and susceptibility to other infections.

Some limitations should be considered when interpreting the results of this study. Firstly, the cross-sectional design provides a snapshot of pathogen circulation at a specific time point and does not capture seasonal variations in tick abundance or pathogen occurrence. This aspect is particularly relevant because tick activity in Mediterranean regions is strongly influenced by temperature, humidity, and host availability [19].

Another limitation concerns the molecular characterization of some detected microorganisms. The short length of certain PCR amplicons limited species-level identification and prevented complete genetic characterization for some agents. Future studies using multilocus sequencing approaches or next-generation sequencing could provide a more detailed understanding of pathogen diversity and genetic relationships among strains circulating in Sardinian livestock systems.

## 5. Conclusions

Despite these limitations, this cross-sectional molecular survey provides valuable information on the diversity of ticks and tick-borne microorganisms detected in small-ruminant farms in Sardinia. The predominance of *Rhipicephalus* ticks and the detection of bacterial and protozoan DNA in ticks and selected small ruminants highlight the complexity of microorganism–host–vector interactions in Mediterranean pastoral systems. These findings contribute to improving the understanding of the local distribution of tick-associated microorganisms and provide baseline data for future epidemiological investigations. Continued monitoring, combined with integrated surveillance approaches and further studies assessing pathogen transmission dynamics, vector competence, and potential impacts on animal and public health, will be essential to better characterize the epidemiological relevance of tick-borne microorganisms in Sardinian livestock systems.

## Figures and Tables

**Table 1:** Characteristics of the livestock farms included in the study, showing municipality and locality, number of animals sampled for blood collection/herd size, number of ticks collected and analyzed, and livestock species raised.

Municipality	Locality	Animals Sampled/Herd Size (%)	Tested Ticks	Species Raised
Baunei	Forrola	10/215 (4.65%)	77	Goats
Ulassai	Cuccuru e Pau	1/26 (3.85%)	0	Goats
Ulassai	Su Accu	10/130 (7.69%)	18	Goats
Talana	Toce dorgo	8/341 (2.35%)	33	Goats
Talana	Su Nercone	10/422 (2.37%)	23	Goats
Urzulei	Su Sogadoro	7/243 (2.88%)	30	Goats
Perdasdefogu	Alburuaccesu	6/198 (3.03%)	28	Goats
Ulassai	Su Campu	5/308 (1.62%)	43	Goats
Villagrande Strisaili	Coile brugiau	0/255 (0.00%)	7	Goats
Baunei	Coile Andria	0/80 (0.00%)	2	Goats
Arzana	Usacci	0/305 (0.00%)	8	Goats
Ussassai	Paese	10/66 (15.15%)	69	Sheep
Castiadas	Buddoi	1/66 (1.52%)	4	Sheep
Villaputzu	Quirra	1/199 (0.50%)	1	Sheep
Baunei	Salbene	6/136 (4.41%)	13	Sheep
Castiadas	Brebiscios	0/400 (0.00%)	7	Sheep
Villagrande Strisaili	Sa Sorgargia	0/259 (0.00%)	1	Sheep
**TOT**		75/3649 (2.06%)	364	

**Table 2:** Primers and probes used for the molecular detection of tick-borne pathogens. The table reports the target gene, primer/probe sequences, PCR assay (real-time or conventional PCR), expected amplicon size, and corresponding reference for each assay.

Bacterial	Gene Target	Primer/Probe	PCR Tool	Sequence (5′–3′)	Amplicon Size (bp)	Ref.
*Rickettsia* spp.	*gltA*	CS5-F	Rt-PCR	GAGAGAAAATTATATATCCAAATGTTGAT	147	[20]
		CS6-R		AGGGTCTTCGTGCATTTCTT		
		CS5-FAM		CATTGTGCCATCCAGCCTACGGT		
		gltA-F	Standard PCR	CCTATGGCTATTATGCTTGC	770	[21]
		gltA-R		ATTGCAAAAAGTACAGTGAACA		
*Babesia* spp./*Theileria* spp.	5.8S rRNA	5.8S-F	Rt-PCR	AYYKTYAGCGRTGGATGTC		[22]
		5.8S-R		TCGCAGRAGTCTKCAAGTC	84	
		5.8S-FAM		TTYGCTGCGTCCTTCATCGTTGT		
	18S rRNA	BJ1-F	Standard PCR	GTCTTGTAATTGGAATGATGG	452	[23]
		BJ1-R		TAGTTTATGGTTAGGACTACG		
*Coxiella* spp.	IS1111	Cox-F	Rt-PCR	GTCTTAAGGTGGGCTGCGTG	295	[24]
		Cox-R		CCCCGAATCTCATTGATCAGC		
		Cox-P-FAM		AGCGAACCATTGGTATCGGACGTT		
	16s rRNA	Cox16S-F	Standard PCR	TGAGAACTAGCTGTTGGRRAGT	624	[25]
		Cox16S-R		GCCTACCCGCTTCTGGTACAATT		
*Anaplasma* spp.	16s rRNA	HGE–521F	Standard PCR	TGTAGGCGGTTCGGTAAGTTAAAG	293	[26]
		HGE–790R		CTTAACGCGTTAGCTACAACACAG		

**Table 3:** PCR detection of tick-borne pathogens in goats and sheep from sampled farms in Sardinia. Values are reported as positive animals/total tested animals (*n*/N) and percentage positivity (%).

		PCRs			
Municipality	Sampled Animals/Animals in Farm	*Rickettsia* spp. n/N (%)	*Coxiella* spp. n/N (%)	*Anaplasma* spp. n/N (%)	Piroplasms n/N (%)
Goat Farms					
Baunei	10/215	0/10 (0)	0/10 (0)	0/10 (0)	2/10 (20.0)
Ulassai	1/26	0/1 (0)	0/1 (0)	0/1 (0)	0/1 (0)
Ulassai	10/130	0/10 (0)	0/10 (0)	0/10 (0)	0/10 (0)
Talana	8/341	0/8 (0)	0/8 (0)	2/8 (25.0)	0/8 (0)
Talana	10/422	0/10 (0)	0/10 (0)	2/10 (20.0)	0/10 (0)
Urzulei	7/243	0/7 (0)	0/7 (0)	3/7 (42.9)	0/7 (0)
Perdasdefogu	6/198	0/6 (0)	0/6 (0)	4/6 (66.7)	0/6 (0)
Ulassai	5/308	0/5 (0)	0/5 (0)	5/5 (100)	0/5 (0)
**Total**	**57/1883**	**0/57 (0)**	**0/57 (0)**	**16/57 (28.1)**	**2/57 (3.5)**
Sheep Farms					
Ussassai	10/66	0/10 (0)	0/10 (0)	0/10 (0)	7/10 (70.0)
Castiadas	1/66	0/1 (0)	0/1 (0)	0/1 (0)	1/1 (100)
Villaputzu	1/199	0/1 (0)	0/1 (0)	1/1 (100)	0/1 (0)
Baunei	6/136	0/6 (0)	0/6 (0)	6/6 (100)	6/6 (100)
**Total**	**18/467**	**0/18 (0)**	**0/18 (0)**	**7/18 (38.9)**	**14/18 (77.8)**
**Overall total**	**75/2350**	**0/75 (0)**	**0/75 (0)**	**23/75 (30.7)**	**16/75 (21.3)**

**Table 4:** Molecular detection by Rt-PCRs of tick-borne pathogens in ticks collected from small ruminants in Sardinia. Results are expressed as positive ticks/total tested ticks (n/N) and percentage positivity (%). *Coxiella* spp. analyses were performed on 346 ticks due to unavailable DNA from 18 specimens (nd, not determined).

			Rt-PCRs			
Farms	N° Ticks	Tick Species	Piroplasma spp. n/N (%)	*Coxiella* spp. n/N (%)	*Rickettsia* spp. n/N (%)	*Anaplasma* spp. n/N (%)
Baunei	77	*R. sanguineus*	2/77 (2.6)	77/77 (100)	50/77 (64.9)	12/77 (15.6)
Ussassai	68	*R. sanguineus*	20/68 (29.4)	2/68 (2.9)	21/68 (30.9)	18/68 (26.5)
	1	*R. bursa*	0/1 (0)	0/1 (0)	0/1 (0)	0/1 (0)
Ulassai	18	*R. bursa*	2/18 (11.1)	0/18 (0)	5/18 (27.8)	0/18 (0)
Castiadas	4	*R. sanguineus*	1/4 (25.0)	nd	0/4 (0)	0/4 (0)
Talana	16	*R. sanguineus*	2/16 (12.5)	0/16 (0)	1/16 (6.3)	1/16 (6.3)
	17	*R. bursa*	1/17 (5.9)	0/17 (0)	2/17 (11.8)	4/17 (23.5)
Talana	9	*R. sanguineus*	1/9 (11.1)	0/9 (0)	0/9 (0)	1/9 (11.1)
	14	*R. bursa*	0/14 (0)	0/14 (0)	0/14 (0)	0/14 (0)
Villaputzu	1	*R. bursa*	0/1 (0)	0/1 (0)	0/1 (0)	0/1 (0)
Urzulei	20	*R. sanguineus*	0/20 (0)	0/20 (0)	0/20 (0)	3/20 (15.0)
	9	*R. bursa*	0/9 (0)	0/9 (0)	0/9 (0)	3/9 (33.3)
	1	*R. turanicus*	0/1 (0)	0/1 (0)	0/1 (0)	1/1 (100)
Perdasdefogu	22	*R. sanguineus*	0/22 (0)	0/22 (0)	1/22 (4.5)	4/22 (18.2)
	5	*R. bursa*	4/5 (80.0)	0/5 (0)	3/5 (60.0)	2/5 (40.0)
	1	*R. pusillus*	0/1 (0)	0/1 (0)	0/1 (0)	1/1 (100)
Baunei	13	*R. sanguineus*	0/13 (0)	1/13 (7.7)	0/13 (0)	3/13 (23.1)
Ulassai	5	*R. sanguineus*	1/5 (20.0)	0/5 (0)	0/5 (0)	0/5 (0)
	38	*R. bursa*	7/38 (18.4)	2/38 (5.3)	0/38 (0)	13/38 (34.2)
Castiadas	7	*R. bursa*	0/7 (0)	nd	1/7 (14.3)	0/7 (0)
Villagrande Strisaili	6	*R. sanguineus*	1/6 (16.7)	nd	4/6 (66.7)	1/6 (16.7)
	1	*H. sulcata*	0/1 (0)	nd	1/1 (100)	0/1 (0)
Baunei	2	*R. sanguineus*	0/2 (0)	0/2 (0)	0/2 (0)	0/2 (0)
Arzana	8	*R. sanguineus*	1/8 (12.5)	0/8 (0)	0/8 (0)	0/8 (0)
Villagrande Strisaili	1	*R. sanguineus*	0/1 (0)	0/1 (0)	0/1 (0)	0/1 (0)
**Total **	**364**		43/364 (11.8)	82/346 (23.7)	94/364 (25.8)	67/364 (17.6)

**Table 5:** Distribution of *Anaplasma*-positive ticks among sampled animals. The table reports positive tick species, number of *Anaplasma*-positive ticks/total ticks collected, and engorgement status. Grey-highlighted rows indicate animals positive for *Anaplasma* spp. and associated with positive ticks. NI: non-engorged; I: engorged.

Id Animal	Animal Species	Positive Tick Species	*Anaplasma*-Positive Ticks/Total Ticks Collected	Engorged Status of Positive Ticks
2	goat	*R. sanguineus*	2/11	NI
3	goat	*R. sanguineus*	2/14	NI
4	goat	*R. sanguineus*	2/10	1 NI; 1 I
5	goat	*R. sanguineus*	1/3	NI
6	goat	*R. sanguineus*	1/2	NI
7	goat	*R. sanguineus*	3/5	2 NI; 1 I
10	goat	*R. sanguineus*	1/9	NI
11	sheep	*R. sanguineus*	2/5	1 NI; 1 I
13	sheep	*R. sanguineus*	1/3	NI
14	sheep	*R. sanguineus*	1/18	I
15	sheep	*R. sanguineus*	2/3	I
17	sheep	*R. sanguineus*	6/11	2 NI; 4 I
18	sheep	*R. sanguineus*	1/8	NI
19	sheep	*R. sanguineus*	1/3	I
20	sheep	*R. sanguineus*	4/9	NI
38	goat	*R. bursa*	1/3	I
		*R. sanguineus*	1/3	I
39	goat	*R. bursa*	3/12	I
45	goat	*R. sanguineus*	1/1	NI
55	goat	*R. bursa*	2/4	1 NI; 1 I
56	goat	*R. turanicum*	1/2	I
58	goat	*R. sanguineus*	2/5	1 NI; 1 I
59	goat	*R. bursa*	1/2	NI
61	goat	*R. sanguineus*	1/7	I
62	goat	*R. bursa*	1/1	I
64	goat	*R. sanguineus*	1/2	I
65	goat	*R. pusillus*	1/5	NI
		*R. sanguineus*	2/5	I
66	goat	*R. sanguineus*	1/6	NI
67	goat	*R. sanguineus*	1/1	NI
72	goat	*R. sanguineus*	1/7	I
73	sheep	*R. sanguineus*	1/1	NI
75	sheep	*R. sanguineus*	1/1	NI
76	sheep	*R. sanguineus*	1/1	NI
83	goat	*R. bursa*	9/13	I
84	goat	*R. bursa*	2/11	I
85	goat	*R. bursa*	2/5	NI
TOTAL			67/212	

**Table 6:** Distribution of piroplasm-positive ticks collected from sampled small ruminants. The table reports positive tick species, number of piroplasm-positive ticks/total ticks collected, and engorgement status. Grey-highlighted rows indicate animals positive for piroplasms and carrying positive ticks. NI: non-engorged; I: engorged.

Id Animal	Animal Species	Positive Tick Species	Piroplasm-Positive Ticks/Total Ticks Collected	Engorged Status of Positive Ticks
3	goat	*R. sanguineus*	1/14	NI
9	goat	*R. sanguineus*	1/4	NI
11	sheep	*R. sanguineus*	3/5	1 NI; 2 I
12	sheep	*R. sanguineus*	3/4	1 NI; 2 I
14	sheep	*R. sanguineus*	2/18	I
15	sheep	*R. sanguineus*	3/3	1NI; 2 I
16	sheep	*R. sanguineus*	2/5	I
17	sheep	*R. sanguineus*	7/11	4 NI; 3 I
22	goat	*R. bursa*	1/6	NI
28	goat	*R. bursa*	1/2	NI
32	sheep	*R. sanguineus*	1/4	NI
33	goat	*R. bursa*	1/6	NI
36	goat	*R. bursa*	1/2	NI
39	goat	*R. sanguineus*	1/12	I
48	goat	*R. sanguineus*	1/2	I
65	goat	*R. bursa*	1/5	I
70	sheep	*R. bursa*	3/7	NI
72	goat	*R. sanguineus*	1/6	I
82	goat	*R. sanguineus*	1/5	I
83	goat	*R. bursa*	5/13	I
86	goat	*R. sanguineus*	1/5	NI
87	goat	*R. bursa*	2/8	1 NI; 1 I
TOTAL			43/147	

## Data Availability

The original contributions presented in this study are included in the article. Further inquiries can be directed to the corresponding author(s).
